# Efficacy and immune modulation associated with the addition of IMiDs to Daratumumab backbone in multiple myeloma patients refractory to both drug classes: resetting synergistic activity

**DOI:** 10.1038/s41408-024-00988-x

**Published:** 2024-02-06

**Authors:** Ioannis V. Kostopoulos, Despina Fotiou, Maria Gavriatopoulou, Pantelis Rousakis, Ioannis Ntanasis-Stathopoulos, Chrysanthi Panteli, Panagiotis Malandrakis, Magdalini Migkou, Nikolaos Angelis, Nikolaos Kanellias, Evangelos Eleutherakis-Papaiakovou, Foteini Theodorakakou, Maria Krevvata, Evangelos Terpos, Meletios-Athanasios Dimopoulos, Ourania Tsitsilonis, Efstathios Kastritis

**Affiliations:** 1 Flow Cytometry Unit, Department of Biology, School of Science, National and Kapodistrian University of Athens, Athens, Greece; 2 Department of Clinical Therapeutics, School of Medicine, National and Kapodistrian University of Athens, Athens, Greece; 3 Janssen Research & Development, Spring House, PA USA

**Keywords:** Immunotherapy, Myeloma, Tumour immunology, Cancer immunotherapy, Cancer microenvironment

## To the Editor:

Currently, there are several treatment options for patients with a relapsed/refractory Multiple Myeloma (RRMM) status, but the choice of the optimal salvage treatment can be challenging [1]. Following an individualized decision-making approach in MM, each patient’s specific characteristics should be considered, including baseline prognostic factors, age, prior line(s) of therapy with all relevant information regarding toxicities and responses, current performance status and comorbidities [2]. Daratumumab (Dara) and Dara-based regimens are very often the treatment of choice for patients refractory to immunomodulatory agents (IMiDs) and/or proteasome inhibitors (PIs). On the other hand, refractoriness to Dara is associated with poor prognosis and limited subsequent therapeutic potential [3, 4]; hence, treatment of triple-class refractory patients remains a challenge.

**Fig. 1:**
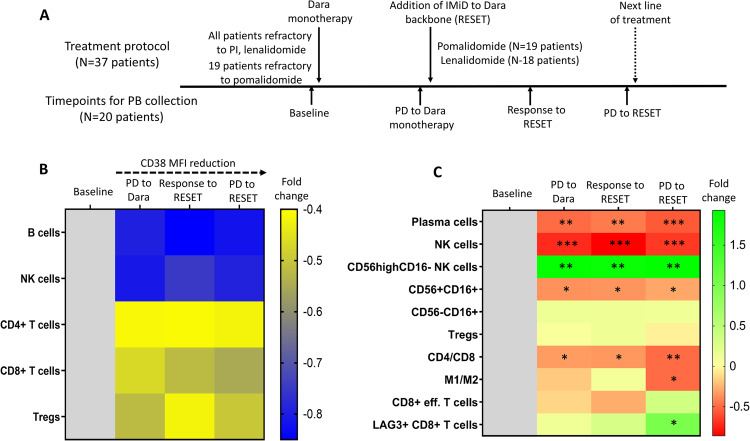
Treatment protocol and phenotypic kinetics during the RESET therapy. **A** Graphical representation of the treatment protocol and the timepoints selected for the analysis of patients’ peripheral blood immune profile. **B** Heatmap showing the CD38 mean fluorescence intensity (MFI) expression reduction due to Dara administration throughout the treatment period. Axis on the right depicts the MFI fold-change of CD38 expression on each immune subset when compared with the baseline levels. **C** Heatmap showing the relevant changes in the prevalence of various immune subsets during the treatment period. Color gradient highlights the fold-change differences in the abundance of each subset at any timepoint when compared with the baseline levels. # calculated as (%) of total nucleated cells ¥ calculated as (%) of total natural killer (NK) cells, € calculated as (%) of total CD8 + T cells, Dara daratumumab, eff. effector, IMiD immunomodulatory agent, PB peripheral blood, PD progressive disease, PI proteasome inhibitor, Tregs regulatory T cells. *p < 0.5; **p < 0.01; ***p < 0.001.

There is limited data supporting that Dara re-treatment with a Dara/IMiD combination may provide benefit, even when prior resistance to both individual drug classes has been developed [5, 6]. Different hypotheses have been proposed for this Dara/IMiD synergy, which, among others, involve an increase in both the T cell- or natural killer (NK) cell-mediated antimyeloma activity, but also the elimination of CD38-mediated checkpoint resistance mechanisms [7]. In the present study, we have evaluated the Dara/IMiD combination in 37 patients who were firstly refractory to at least one PI and one IMiD, and had then progressed on Dara monotherapy which was administered as salvage therapy (i.e., they all were triple-class refractory). Based on the study protocol (Fig. 1A), the last IMiD to which each patient was refractory (pomalidomide in 51% and lenalidomide in 49% of patients) was added, without modulating the Dara backbone (RESET). In parallel, using multiparameter flow cytometry for the discrimination of various immune subsets (Supplementary Tables 1, 2 and Supplementary Figs. 1, 2), we have prospectively assessed patients’ immune profiles at distinct timepoints, to monitor for the dynamics of the resulting immunomodulation during the course of the disease.

The clinical characteristics of the patients are shown in Supplementary Table 3. The median duration of Dara monotherapy was 7.9 months (range 1–38 months), which resulted in an overall response rate (ORR) of 57% [one patient (3%) achieved complete response (CR), 32% of patients very good partial response (VGPR) and 22% partial response (PR)] and a median progression-free survival (PFS) of 7.5 months (range 1–48 months). PFS was associated with the type of response [median PFS: 15, 7 and 2.5 months for patients achieving VGPR or better, PR and minor response (MR) or less, respectively; *p* < 0.0001; Supplementary Fig. 3], but was not affected by baseline prognostic factors and/or the number or type of previous treatments, including autologous stem cell transplantation (ASCT) (Supplementary Table 4).

Dara monotherapy resulted in significant alterations in patients’ peripheral blood (PB) immune profiling that remained stable throughout the study period (Fig. 1B, C). In particular, the expression of CD38 showed a significant decrease in all major populations, even in those with low baseline levels (Fig. 1B). Moreover, Dara administration lead to an unequivocal decrease of total NK cells (*p* < 0.0001) and a significantly skewed CD4/CD8 ratio due to the noticeable increase in the percentage of circulating CD8+ cytotoxic T cells (*p* < 0.01), in agreement with previously reported findings [8, 9]. At subset level, we observed a relative increase in the percentage of the CD56^high^CD16- subset among total NK cells, at the expense of mature CD56+CD16+NK cells (*p* < 0.01; Supplementary Fig. 4). On the other hand, no significant changes were observed in the relative frequencies of regulatory T cells (Tregs) or the M1/M2 ratio (Fig. 1C).

**Fig. 2:**
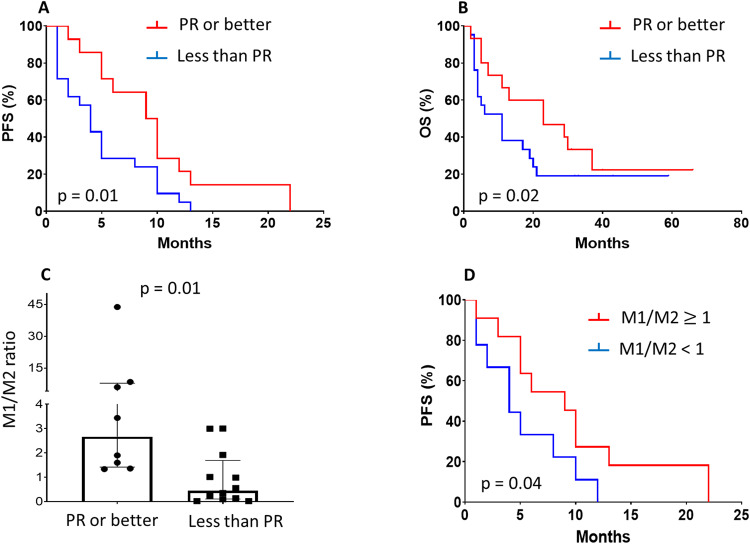
Clinical associations of patients' outcome to RESET therapy. Progression free survival (PFS) (**A**) and overall survival (OS) (**B**) according to therapeutic response to RESET therapy. Association of the baseline M1/M2 ratio with the type of response (**C**) and PFS (**D**) to RESET therapy. PR partial response.

The addition of an IMiD to the Dara backbone proved of clinical relevance, since more than half of the patients showed evidence of response. In specific, over a median duration of 5.5 months at RESET (range: 0.5–24 months), 8.1% (*n* = 3) of patients achieved VGPR, 32.4% (*n* = 12) PR and 13.5% (*n* = 5) MR, whereas 32.4% (*n* = 12) had stable disease (SD) and 13.5% (*n* = 5) showed progressive disease (PD). The median PFS and OS was 9 and 23 months, respectively for patients achieving at least PR at RESET, whereas the median PFS and OS for those showing MR or worse was 4 and 11 months, respectively (HR for PFS: 0.39, 95% CI: 0.18–0.85; *p* = 0.01; HR for OS HR: 0.6, 95% CI: 0.28–1.2; *p* = 0.02; Fig. 2A, B). To date, after a median follow-up of 12 months post RESET initiation (range 2–66 months), all patients have progressed and 8/37 (21.6%) patients are alive and continue with other treatments. Of note, the RESET outcomes were independent of the dose or type of the IMiD used, prior response status to Dara monotherapy, number of prior treatments (more or less than 3), ISS at diagnosis or at the time of RESET initiation (Supplementary Table 5).

The addition of an IMiD at RESET did not cause additional immune profile alterations to those caused by Dara monotherapy (Fig. 1C). Even between the timepoints of response and subsequent progression to RESET (*N* = 19), the relative frequency of the various immune subsets remained stable with minor fluctuations. The proportion of LAG3+CD8+T cells showed a slight but steady increase post RESET initiation, which for some patients was more pronounced at the time of progression to RESET, probably reflecting a gradual development of T cell exhaustion over the course of therapy. On clinical grounds, we examined for possible associations between the levels of each immune subset prior to RESET with the subsequent clinical outcome. The most important predictor of response to RESET therapy proved to be the M1/M2 ratio. In particular, those patients achieving a PR or better to RESET had a median M1/M2 ratio of 2.7 vs. a median ratio of 0.4 for the non-responsive counterpart (*p* = 0.01; Fig. 2C), whereas in terms of PFS, those patients with a M1/M2 value of higher or equal to 1, had a 2-fold lower risk to a subsequent progression (HR: 0.5, 95% CI: 0.2–1.0; *p* = 0.04; Fig. 2D).

Based on its significant efficacy, Dara has been increasingly used as part of first line therapy over the last years. Therefore, its utilization at subsequent lines remains obscure, and little is known about its efficacy when applied as a re-treatment option in previously exposed and refractory patients. The results of this study highlight that Dara re-treatment strategy via the addition of an IMiD is feasible and provides respectable response rates and PFS in triple refractory patients. Interestingly, the type of response to RESET therapy did not correlate with previous responses to either Dara monotherapy or the last IMiD used, suggesting a robust synergistic effect of Dara plus IMiD that stands independently from responses to each agent alone. Moreover, we observed a similar clinical benefit between patients receiving pomalidomide or lenalidomide at RESET, a fact that implies a similar level of the combinational synergy irrespective of the type of IMiD used.

Current therapeutic approaches in MM target both at the elimination of clonal cells, but also at the modification of the immunosuppressive bone marrow (BM) niche, and the disruption of the interactions between myeloma cells and their microenvironment that promotes tumor cell growth and proliferation [10]. In this regard, Dara has potent anti-myeloma activity, as besides the direct Fc-dependent killing mechanisms, it also modifies the tumor microenvironment through the elimination of CD38-expressing immune populations [7, 8]. Our results showed significant immune profiling changes due to Dara administration sustained throughout the whole study period. These observations, together with the consistently reduced levels of CD38 in all immune populations, suggest a perpetual immune modulation within a modified BM niche (due to Dara administration), which does not alter its composition upon the addition of the IMiD. Therefore, the responses observed to the RESET combination could be possibly attributed to the anti-myeloma effects of IMiDs that become more active in the new Dara-induced microenvironmental setting.

At subset level, we observed a relative increase in the prevalence of the CD56^high^CD16- NK cell subset, a group of NK cells with significant immunomodulatory potential via the production of various cytokines [11]. A recent study by Viola et al. [12], showed that targeting CD38 on NK cells is essential for Dara-induced immunomodulation and Dara treatment led to significantly reduced CD16 expression on NK cells, observations which overall agree with our findings. On clinical grounds, macrophage polarization towards the M1 phenotype correlated with favorable patients’ outcome, verifying similar results by Chen et al in a newly-diagnosed MM setting [13]. The increased M1/M2 ratio may correlate with both a direct M1-mediated cytotoxic effect against myeloma cells and/or a reduced M2-directed immunosuppression, which may involve the secretion of interleukin-10, tumor growth factor-β and other anti-inflammatory cytokines that sustain myeloma growth and proliferation [14].

In conclusion, our results indicate that in selected patients with RRMM, re-treatment with a combination that retains a Dara backbone, may be associated with clinical benefit. Such regimens may be a bridge therapy, offering a significant disease burden reduction when no other options are available immediately (e.g. prior to CAR-T immunotherapy). Furthermore, our results highlight the dynamics of the immune modulation caused by Dara and suggest that the immune microenvironment of the BM (partly reflected in PB) [15] may play an important role on the efficiency of the Dara/IMiD synergistic effect.

### Supplementary information

Supplementary inforamtion

## Data Availability

The results of the study are available upon reasonable request from the corresponding author.
