# Supplementary material for: Efficacy and immune modulation associated with the addition of IMiDs to Daratumumab backbone in multiple myeloma patients refractory to both drug classes: resetting synergistic activity
Source: Blood Cancer J. 2024 Feb 6;14(1):26. doi: 10.1038/s41408-024-00988-x (PMC10847500; doi:10.1038/s41408-024-00988-x)
Supplement: Supplementary file 1 — Supplementary inforamtion [file 41408_2024_988_MOESM1_ESM.pdf]

Supplementary Table 1. Multiparameter panels used for the analysis of immune profiling with flow cytometry

	**FITC**	**PE**	**PeCy7**	**PerCPCy5.5**	**APC**	**APCCy7**	**BV421**	**BV510**
**Panel 1**	CD38	Granzyme B	CD127	7-AAD	CD25	CD4	LAG-3	CD3
**Panel 2**	CD14	CD56	CD66b	7-AAD	CD80	CD45	CD16	CD3/CD19

Supplementary Table 2. Immune subsets and the relative phenotypic strategy used for their discrimination

**Immune subset**	**Markers' expression**
All nucleated cells	$7AAD^{-}$ $CD45^{+}$
Plasma cells	$CD38^{++}$ $CD138^{+}$
T cells	$CD3^{+}$ SSC^{low} FSC^{low}
CD4+ T cells	$CD3^{+}$ $CD4^{+}$ SSC^{low} FSC^{low}
CD8+ T cells	$CD3^{+}$ $CD4^{-}$ SSC^{low} FSC^{low}
T regulatory cells (Tregs)	$CD3^{+}$ $CD4^{+}$ $CD25^{+}$ $CD127^{low}$ SSC^{low}
Activated CD8+ T cells	$CD3^{+}$ $CD4^{-}$ Granzyme B^{+} SSC^{low} FSC^{low}
Exhausted CD8+ T cells	$CD3^{+}$ $CD4^{-}$ $Lag3^{+}$ SSC^{low} FSC^{low}
NK cells	Lin^{-} $CD56^{+}$ SSC^{low} FSC^{low} & Lin^{-} $CD56^{-}$ $CD16^{+}$ SSC^{low} FSC^{low}
$CD56^{high}$ $CD16^{-}$ NK cells	Lin^{-} $CD56^{++}$ $CD16^{-}$ SSC^{low} FSC^{low}
$CD56^{+}$ $CD16^{+}$ NK cells	Lin^{-} $CD56^{+}$ $CD16^{+}$ SSC^{low} FSC^{low}
$CD56^{-}$ $CD16^{+}$ NK cells	Lin^{-} $CD56^{-}$ $CD16^{+}$ SSC^{low} FSC^{low}
M1 monocytes	Lin^{-} $CD56^{-}$ $CD66b^{-}$ $CD14^{+}$ $CD16^{+}$ $CD80^{+}$
M2 monocytes	Lin^{-} $CD56^{-}$ $CD66b^{-}$ $CD14^{+}$ $CD16^{+}$ $CD80^{-}$

Abbreviations: Lin: Lineage for CD3 & CD19; SSC: Side scatter; FSC: Forward scatter

Supplementary Table 3. Patient and treatment characteristics at baseline, after daratumumab monotherapy and at RESET

Clinical characteristics	Patients (n=37)
**Baseline (prior to Dara monotherapy)**	
Age in years, median (range)	73 (52-86)
Male gender (%)	66
ECOG PS, median	1
ISS stage I/II/III (%)	30 / 23 / 47
R-ISS stage I/II/III (%)	32 / 47 / 21
Heavy chain IgG/IgA/IgM (%)	29 / 53 / 12
Light chain κ/λ (%)	65 / 35
Prior Tx lines, median (range)	3 (2-16)
Prior ASCT (%)	38
Prior Tx lines<br>Lenalidomide, exposed/refractory (%)<br>Pomalidomide, exposed/refractory (%)<br>Bortezomib, exposed/refractory (%)<br>Carfilzomib, exposed/refractory (%)<br>Ixazomib, exposed/refractory (%)<br>PI (total), exposed/refractory<br>Alkylating agent, exposed/refractory (%)	 100 / 100<br>51 / 51<br>100 / 67<br>27 / 27<br>22 / 18<br>100 /100<br>59 / 59
**Daratumumab monotherapy**	
Duration in months, median (range)	7.9 (1-38)
ORR (≥PR) (%)<br>CR/VGPR/PR (%)	57<br>3 / 32 / 22
Months since last IMiD Tx, median (range)	20 (1-59)
**RESET**	
IMiD added<br>Lenalidomide/Pomalidomide (%)	 49 / 51
Duration in months, median (range)	5.5 (0.5-24)
ORR (≥PR) (%)<br>CR/VGPR/PR (%)	40<br>0 / 8 / 32
Months from diagnosis to RESET, median (range)	67 (8.0-185)

ASCT, autologous stem cell transplantation; CR, complete response; ECOG PS, Easter Cooperative Oncology Group performance status; IMiD, immunomodulatory agent; ISS, International Staging System; ORR, overall response rate; PI, proteasome inhibitor; PR, partial response; R-ISS, revised International Staging System; Tx, treatment; VGPR, very good partial response.

Supplementary Table 4. Progression-free survival to daratumumab monotherapy according to risk factors

Risk factor	Risk category	Median PFS (months)	P value
ISS	ISS III vs. ISS I or ISS II	7.5 vs. 7	0.55
Previous lines of treatment	1-3 vs. > 3	7 vs. 9	0.73
Eligibility for ASCT	Yes vs. No	8 vs. 7.5	0.89
Response to daratumumab	CR/VGPR vs. PR vs. MR/SD	15 vs. 7 vs. 2.5	< 0.0001

ISS, International staging system; ASCT, autologous stem cell transplantation; CR, complete remission; VGPR, very good partial response; PR, partial response; MR, minor response; SD, stable disease

Supplementary Table 5. Progression-free survival to RESET according to risk factors

Risk factor	Risk category	Median PFS (months)	P value
Type of IMiD added	Pomalidomide vs. Lenalidomide	4.5 vs. 5	0.38
Prior response to Daratumumab monotherapy	CR/VGPR vs. PR vs. MR/SD	5 vs. 8 vs. 4.5	0.15
Previous lines of treatment	1-3 vs. > 3	5 vs. 9	0.68
ISS at diagnosis	ISS III vs. ISS I or ISS II	6.5 vs. 4	0.33
ISS at the time of RESET	ISS III vs. ISS I or ISS II	5 vs. 5	0.24
Response to RESET	CR/VGPR vs. PR vs. MR/SD	15 vs. 7 vs. 2.5	0.01

IMiD, Immunomodulatory drug; ISS, International staging system; CR, complete remission; VGPR, very good partial response; PR, partial response; MR, minor response; SD, stable disease

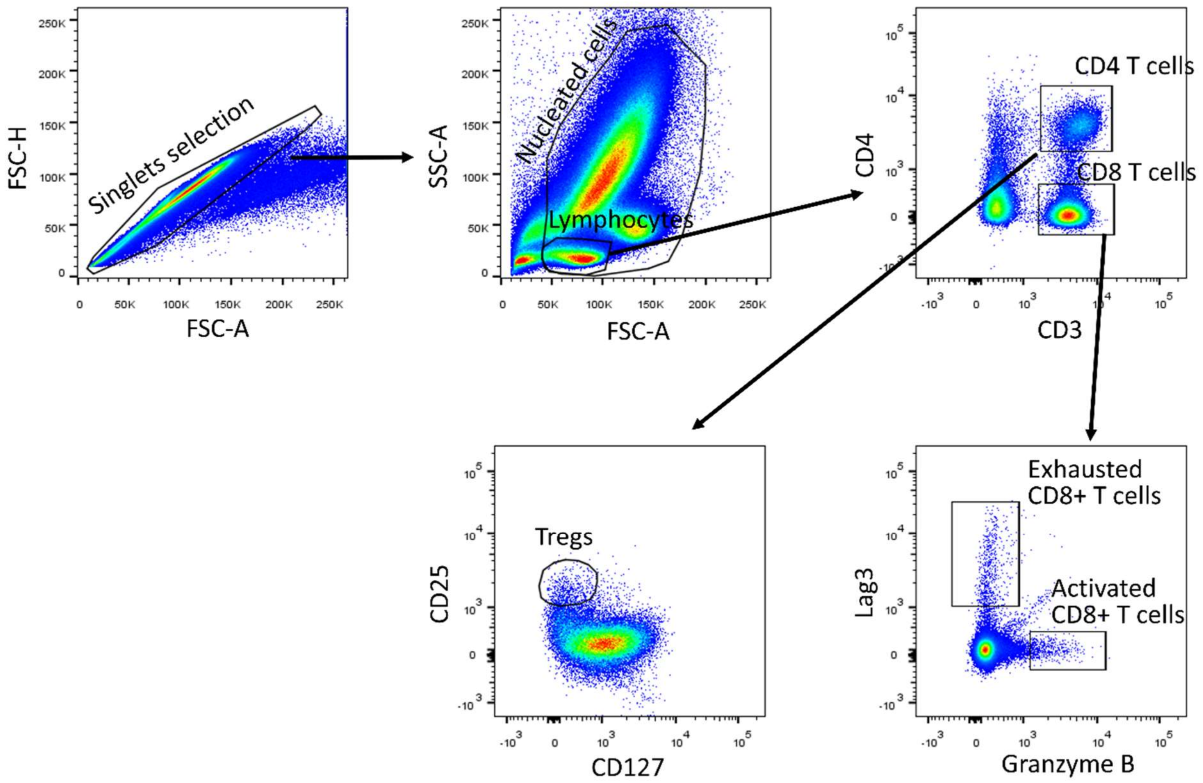

Supplementary Figure 1. Gating strategy followed for the identification of the various T subsets described in the study (*Panel 1*).

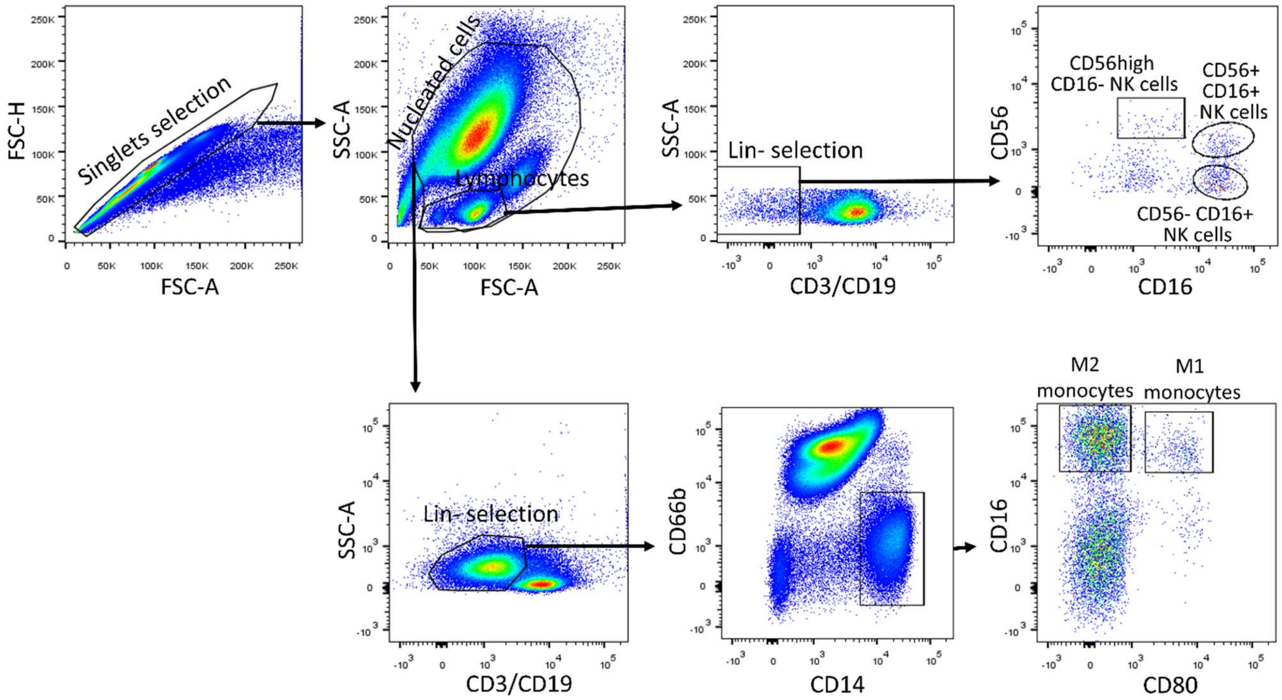

Supplementary Figure 2. Gating strategy followed for the identification of the NK cell subsets and the M1/M2 monocytes described in the study (*Panel 2*).

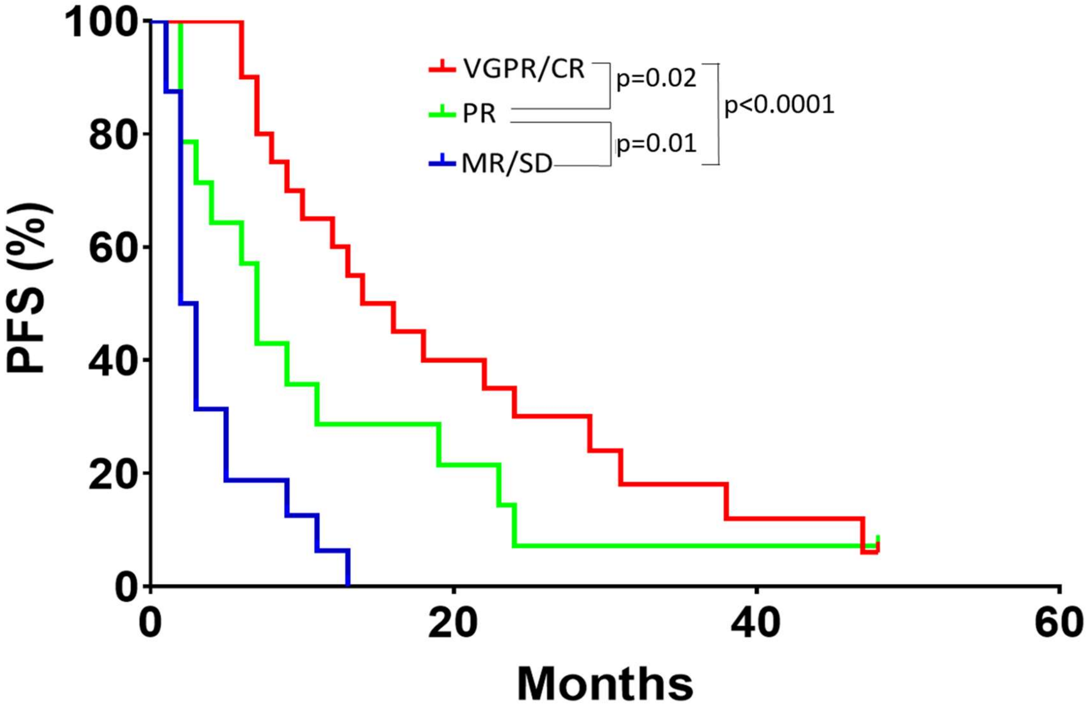

Supplementary Figure 3. Progression free survival (PFS) according to therapeutic response to Dara monotherapy. CR, complete response; MR, minor response; PR, partial response; SD, stable disease; VGPR, very good partial response.

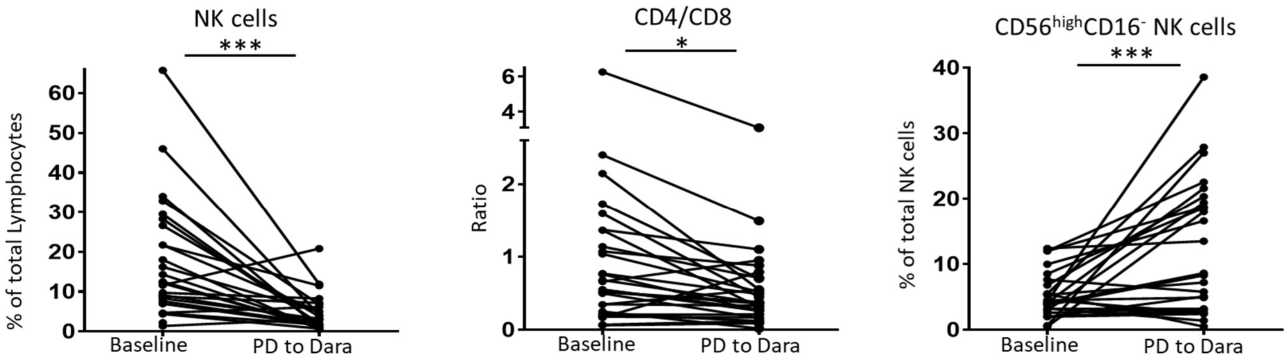

Supplementary Figure 4. Changes in the relative prevalence of NK cells, CD4/CD8 ratio and the CD56^{high}CD16^{-} NK compartment, before and after Dara monotherapy. *, p<0.5; ***, p<0.001.
